# Frequency-Dependent Modulation of Short-Term Neuronal Dynamics in the Female and Male Dorsal and Ventral Rat Hippocampus

**DOI:** 10.3390/ijms26178424

**Published:** 2025-08-29

**Authors:** Athina Miliou, Giota Tsotsokou, Michaela Tsouka, Andriana Koutsoumpa, Costas Papatheodoropoulos

**Affiliations:** 1 Laboratory of Physiology-Neurophysiology, Department of Medicine, University of Patras, 26500 Patras, Greece; athinamhliou@gmail.com (A.M.); giotatsotsokou@gmail.com (G.T.); mihaela.tsouka.42@gmail.com (M.T.); 2 Molecular Systems Biology, Groningen Biomolecular Sciences and Biotechnology Institute, University of Groningen, Nijenborgh 4, 9747 AG Groningen, The Netherlands; andrianakoutsoumpa@gmail.com

**Keywords:** short-term synaptic plasticity, short-term dynamics, synaptic transmission, excitability, gender, sex, septotemporal, dorsoventral, hippocampus, rat

## Abstract

Short-term synaptic plasticity (STSP) and short-term neuronal dynamics (STND) are fundamental properties of neural circuits, essential for information processing and brain function. Emerging evidence suggests that biological sex may influence these properties, yet sex-related differences in STSP and STND remain underexplored. This study investigates sex-specific differences in short-term synaptic plasticity (STSP) and neuronal dynamics (STND) along the dorsoventral axis of the rat hippocampus. Our findings reveal that both STSP and STND exhibit significant variation between female and male subjects. These differences are particularly pronounced in the ventral hippocampus, a region associated with affective and motivational processes. Given the role of short-term activity-dependent neuronal phenomena in modulating information processing and network function, these findings suggest potential functional implications for sex-specific cognitive and emotional regulation. The results highlight the importance of incorporating sex as a biological variable in studies of hippocampal physiology and its relation to behavior.

## 1. Introduction

Short-term synaptic plasticity (STSP) refers to transient forms of activity-dependent changes in synaptic strength enabling several types of real-time information processing, including frequency-dependent filtering, gain control, and adaptive responses to sensory inputs [1,2,3,4,5]. STSP is proposed to be crucial in brain functions such as working memory and decision making [6,7,8,9]. Despite its significance, the majority of existing studies on STSP have been conducted predominantly in male subjects, with limited consideration of female-specific mechanisms, and experimental results obtained from males could be inapplicable to females [10,11,12].

However, emerging evidence reveals sex-specific differences in synaptic plasticity mechanisms at various levels of neural organization; for reviews, see [11,12,13,14,15,16]. For instance, variations in the ability for long-term synaptic potentiation have been shown during the estrous cycle [17,18,19]. Furthermore, estradiol lowers the threshold for the induction of LTP [20,21] and enhances the NMDA receptor-dependent LTP at CA1 hippocampal synapses [22,23]. Additionally, the magnitude of hippocampal long-term potentiation has been found to be higher in males compared with female animals [24,25,26]. Even when a similar magnitude of LTP can be reached in females and males this is mediated by different molecular mechanisms. cAMP-regulated protein kinase is required for LTP in females but not males, and both L-type calcium channels and internal calcium stores appear to be required in females but not males [27]. Interestingly, region-specific sex-related differences have been reported in the hippocampus with the ventral hippocampus of female rats expressing higher levels of estrogen receptors than the dorsal hippocampus [28,29]. These findings could suggest that STSP dynamics may also diverge between males and females in ways that impact neural computations. Indeed, sex-related differences have been described at the molecular, synaptic, and cellular levels that could impact on STSP and behavior. For instance, the nucleus accumbens of females displays larger readily releasable neurotransmitter pools compared to males [30], and medial prefrontal synapses display a higher transmitter release probability in females than males [31]. These characteristics are expected to have a strong impact on STSP properties [1,2]. In addition, estrogens can influence spine density and dendritic arborization that normally fluctuate across the estrous cycle [32,33,34,35,36], thereby shaping STSP properties by altering electrotonic properties and synaptic integration [37,38].

In addition to STSP, which reflects activity-dependent changes at the synaptic level, the dynamics of neuronal firing, referred to as short-term neuronal dynamics (STND), play a critical role in shaping the output of neuronal circuits and thus the overall computational properties of brain networks [39,40,41,42,43]. Thus, STND represents rapid, transient changes in neuronal excitability and firing output that shape how local brain circuits respond to repeated or patterned input [44,45]. It has been recently shown that STND in the hippocampal CA1 field, while partially influenced by STSP, can be determined independently by network-level factors such as synaptic inhibition [46] and regional modulation by neuromodulators [46,47,48,49], supporting the idea that these dynamics are an emergent network property, not just a direct reflection of synaptic function.

Therefore, the dorsal and ventral hippocampus exhibit markedly distinct STND profiles [46] and age-related changes [50], suggesting functional specializations along the septotemporal axis and lifespan that contribute to localized information processing properties within the CA1 field. These dynamics are particularly relevant for short-term encoding of temporal information and may serve to optimize gain control, signal amplification, and temporal filtering of input in hippocampal networks [41,42]. Furthermore, STND can modulate how inputs are transformed into output patterns over short timescales [43], especially during high-frequency oscillatory activity such as theta and gamma rhythms, which are prominent in the hippocampus supporting learning and memory. Alterations in normal STND may have important implications for brain disorders. For instance, abnormal short-term dynamics have been implicated in neuropsychiatric diseases such as autism spectrum disorder and schizophrenia [51,52,53], where they may underlie symptoms like working memory dysfunction and impaired temporal filtering of information.

However, whether STND follows sex-related diversification is not known. Given the hippocampus’ key role in emotion and cognition, and the known sex-related differences in affective and cognitive behaviors, investigating STND in both sexes could reveal important physiological substrates for such differences. In addition, alterations in short-term dynamics have been linked to neuropsychiatric disorders, which often exhibit sex-biased prevalence and symptomatology. Therefore, knowing whether and how STND differs between females and males is crucial for understanding sex-related specializations in information processing and may help explain sex-specific vulnerabilities to brain disorders.

In this study, we aimed to investigate sex-specific differences in STSP and STND across the dorsoventral axis of the rat hippocampus, recording field excitatory synaptic potentials (fEPSPs) and population spikes (PSs) from the CA1 field. We hypothesized that males and females would differ in both forms of short-term plasticity and that these differences would vary with hippocampal region, reflecting distinct contributions to cognitive versus emotional processing, which are associated with the dorsal and ventral hippocampus, respectively [54,55]. By examining frequency-dependent modulation of synaptic and neuronal responses, we sought to uncover sex- and region-dependent properties that may underlie differential hippocampal function and behavioral outcomes. Our results reveal significant sex-dependent and region-specific differences in short-term neuronal dynamics. These findings have important implications for understanding sex-specific mechanisms of information processing and broader brain functions.

## 2. Results

### 2.1. Similar Synaptic Transmission and Neuronal Excitability in Female and Male Rats

**Figure 1:**
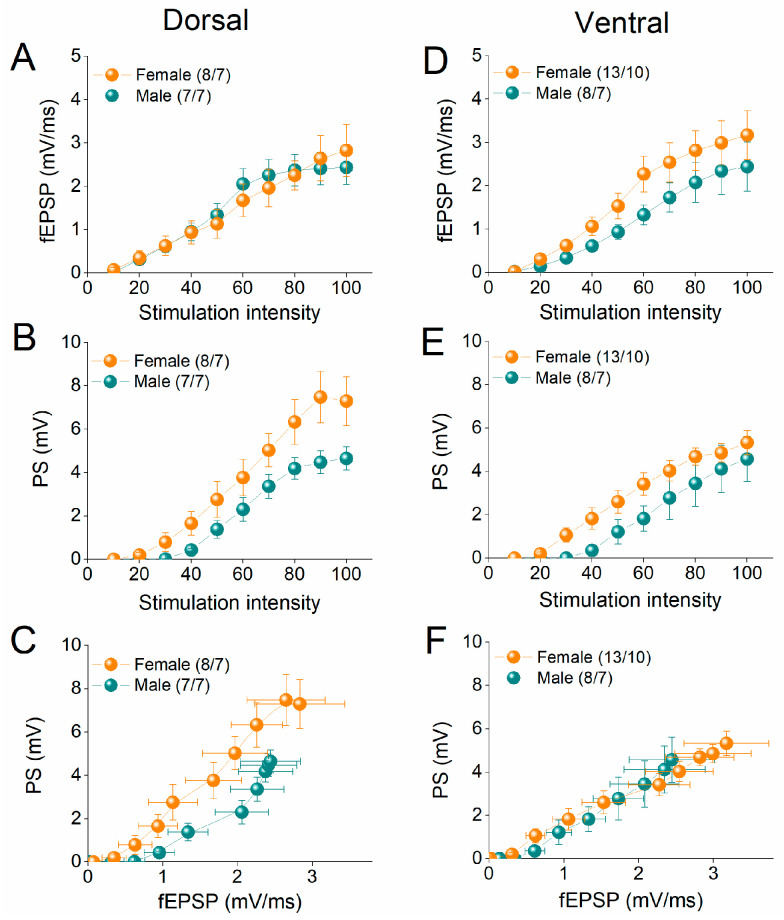
Input–output curves between fEPSP and PS as a function of stimulation current intensity, and between PS and fEPSP, shown for the dorsal hippocampus (**A**–**C**) and the ventral hippocampus (**D**–**F**) of female and male rats. Numbers into parentheses in this and following figures with collective data indicate the number of slices/rats used.

**Figure 2:**
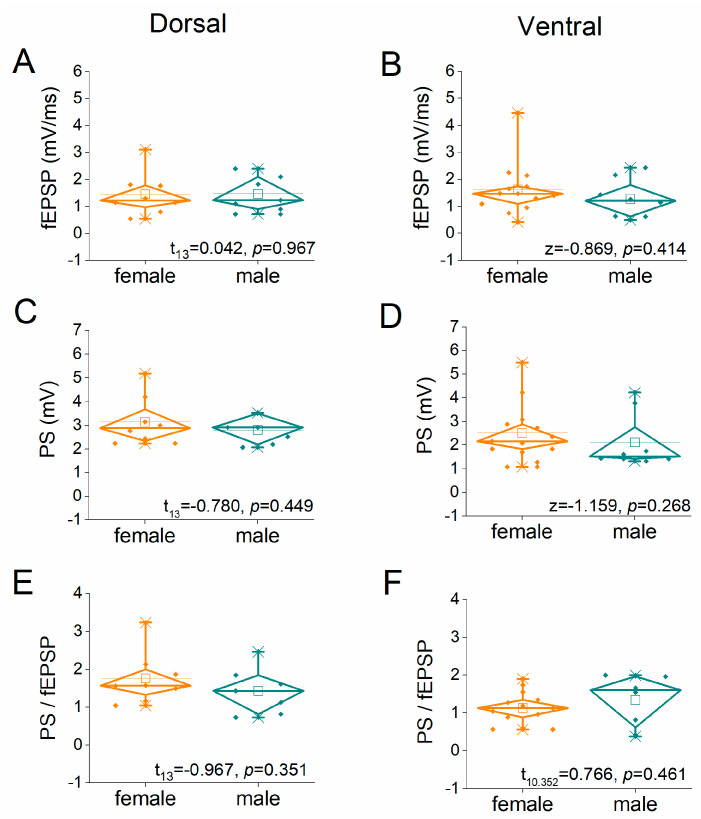
Average values of fEPSP, PS, and PS/fEPSP in the female and male dorsal hippocampus (**A**,**C**,**E**) and ventral hippocampus (**B**,**D**,**F**). Values were derived from the input–output curves presented in Figure 1. The results of independent *t*-tests are shown at the bottom of each graph.

We compared basal excitatory synaptic transmission and neuronal excitability between female and male rats by constructing input–output (I-O) curves relating stimulation current intensity to fEPSP or PS, as well as between fEPSP and PS. Figure 1 shows the I-O relationships for the dorsal and ventral hippocampus in the two sexes. We found that the average fEPSP recorded from either the dorsal or ventral hippocampus did not significantly differ between female and male rats (Figure 2A,B). Furthermore, we examined the excitation between the two sexes in both dorsal and ventral hippocampus. Similarly, our analysis revealed no significant effect of sex on PS either in the dorsal or the ventral hippocampus (Figure 1C,D). In addition, neuronal excitability, assessed by the PS/fEPSP ratio was similar in the female and male dorsal and ventral hippocampus (Figure 1E,F).

### 2.2. Short-Term Synaptic Plasticity (STSP) Differs Between Female and Male Hippocampus

**Figure 3:**
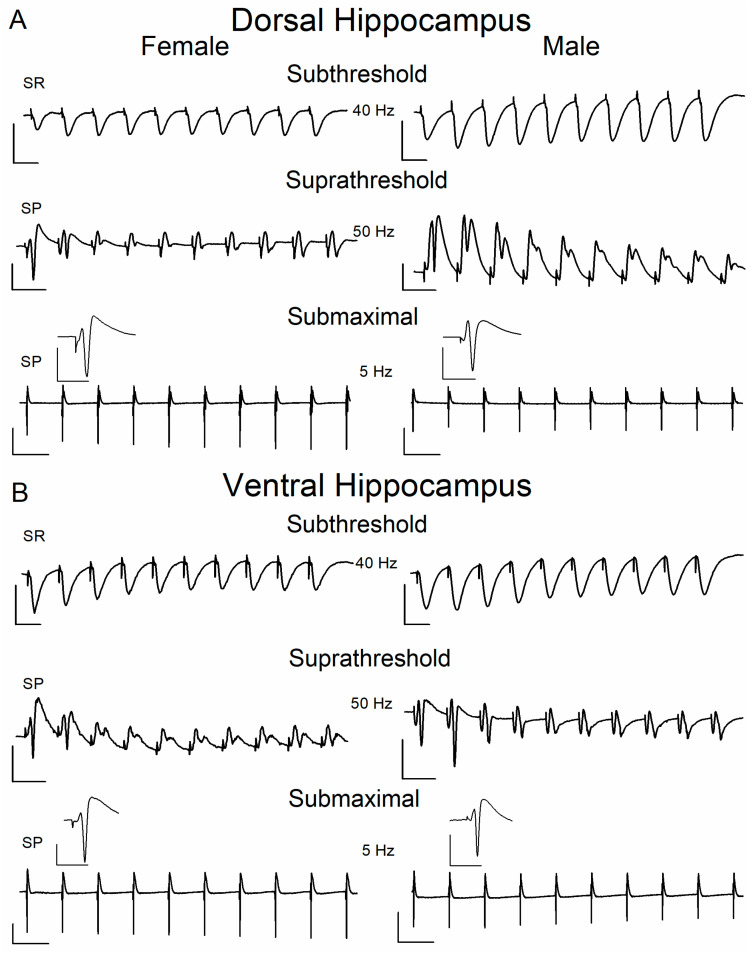
Representative traces of fEPSPs and PS recorded from the CA1 stratum radiatum (SR) and stratum pyramidale (SP), evoked by repetitive stimulation of Schaffer collaterals at the dorsal (**A**) and ventral (**B**) hippocampal slices of female and male rats. Traces illustrate responses to subthreshold stimulation at 40 Hz, suprathreshold stimulation at 50 Hz, and submaximal stimulation at 5 Hz. Calibration bars: 1 mV, 20 ms in traces of 40 Hz and 50 Hz; 3 mV, 200 ms in traces of 5 Hz. Stimulation artifacts are truncated for clarity. In submaximal condition, single PS traces are also presented magnified as inserts; calibration: 3 mV, 10 ms.

To study STSP, we delivered a ten-pulse stimulation train of varying frequency (frequency stimulation) at Schaffer collaterals, and we recorded the fEPSP from the CA1 stratum radiatum using three stimulation current intensities (example recordings are shown in Figure 2). Specifically, we adjusted the stimulation current intensity to alternatively produce: (a) a subthreshold fEPSP (0.4 ± 0.01 mV/ms); (b) a suprathreshold fEPSP (1.47 ± 0.04 mV/ms) that evoked a PS of 1.0–2.0 mV (1.36 ± 0.01 mV); (c) a PS of 75% of its maximum amplitude (4.76 ± 0.1 mV), and a corresponding fEPSP of 2.49 ± 0.05 mV/ms. Examples of responses evoked by frequency stimulation are shown in Figure 3.

**Figure 4:**
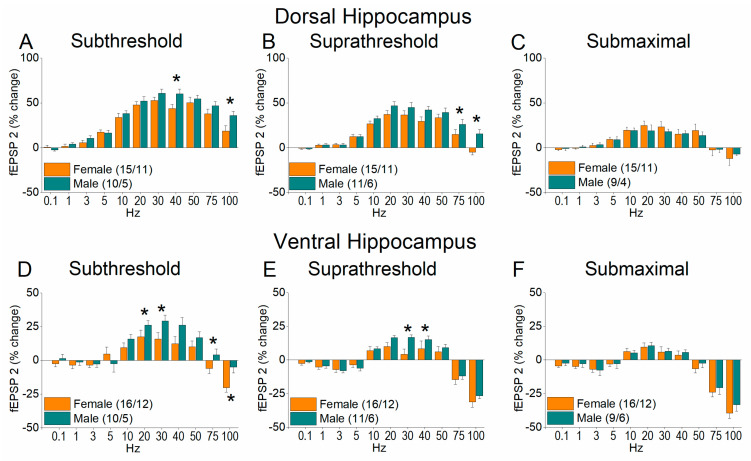
Frequency-dependent modulation of fEPSP in the dorsal hippocampus (**A**–**C**) and ventral hippocampus (**D**–**F**) of female and male rats. Results for the first conditioned response (fEPSP 2) are presented under three stimulation intensities: subthreshold, suprathreshold, and submaximal. Statistically significant differences are denoted by asterisks (independent *t*-test, * *p* < 0.05). Note that both the dorsal and ventral hippocampus of male rats exhibit greater synaptic facilitation than females under subthreshold and suprathreshold, but not submaximal stimulation conditions.

**Figure 5:**
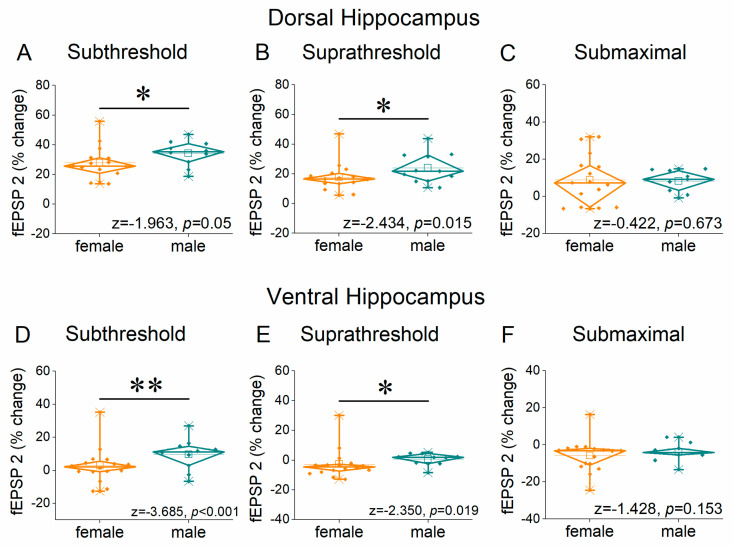
The average percent effects of frequency stimulation on fEPSP 2 are shown for the three stimulation current intensities (subthreshold, suprathreshold, and submaximal). Data are shown for the dorsal hippocampus (**A**–**C**) and the ventral hippocampus (**D**–**F**). Statistically significant differences are denoted by asterisks (independent *t*-test, * *p* < 0.05, ** *p* < 0.01).

Figure 4 and Supplementary Figures S1 and S2 show the results of frequency stimulation on the first conditioned response fEPSP (fEPSP 2) in both the dorsal and ventral hippocampus; this response is equivalent to paired-pulse facilitation. We found that at subthreshold and suprathreshold stimulation, the female dorsal and ventral hippocampus lag behind the male in terms of facilitation of synaptic responses, while at high frequencies of stimulation it shows an absence of facilitation or even depression. More specifically, in the dorsal hippocampus the change in fEPSP 2 significantly differed between females and males at subthreshold (UNIANOVA, *F*_(1, 252)_ = 11.45, *p* < 0.001) and suprathreshold stimulation (*F*_(1, 63)_ = 17.19, *p* < 0.001) but not at submaximal stimulation (UNIANOVA, *F*_(1, 238)_ = 0.17, *p* = 0.733). Similarly, sex-related significant differences were observed at subthreshold (UNIANOVA, *F*_(1, 263)_ = 10.5, *p* < 0.001) and suprathreshold stimulation (*F*_(1, 275)_ = 6.6, *p* < 0.001) but not at submaximal stimulation (UNIANOVA, *F_(_*_1, 252)_ = 1.79, *p* = 0.183). Furthermore, we obtained similar results when comparing the average responses, that is, when pooling together the responses evoked by all stimulation frequencies (Figure 5).

**Figure 6:**
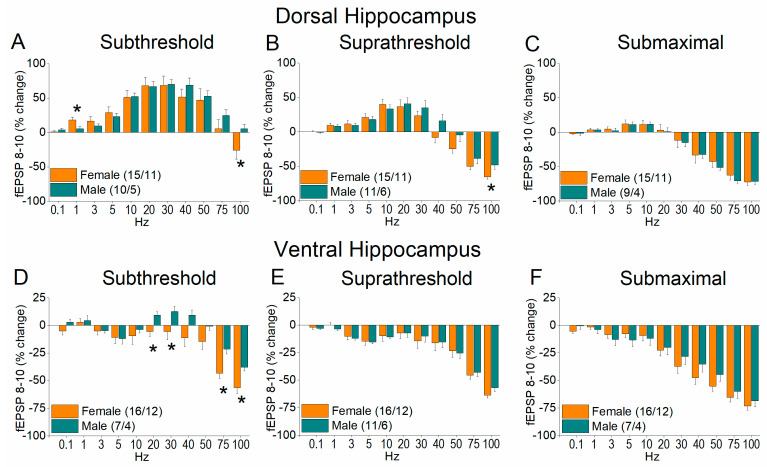
Frequency-dependent modulation of fEPSP in the dorsal hippocampus (**A**–**C**) and ventral hippocampus (**D**–**F**) of female and male rats. Results for the steady state conditioned response (fEPSP 8–10) are presented across three stimulation intensities: subthreshold, suprathreshold, and submaximal. Statistically significant differences are denoted by asterisks (independent *t*-test, * *p* < 0.05). Note that the ventral hippocampus of male rats exhibits greater frequency facilitation or lower frequency depression than females under subthreshold stimulation intensity.

**Figure 7:**
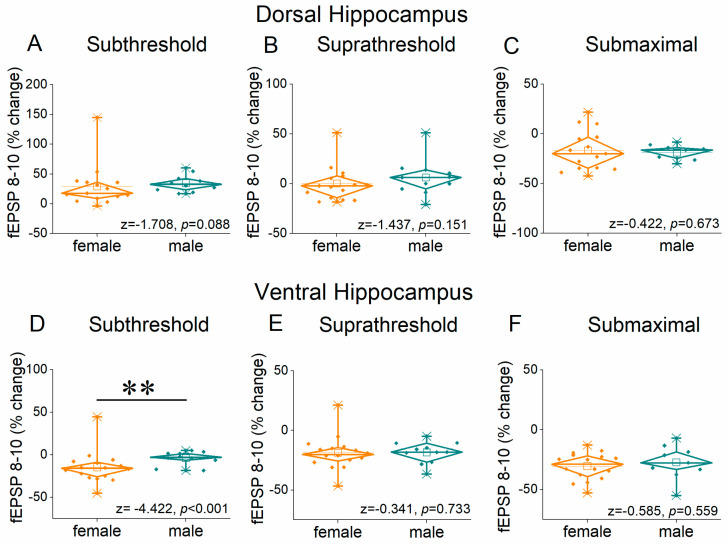
The average percent effects of frequency stimulation on fEPSP 8–10 (steady state) are shown for the three stimulation current intensities (subthreshold, suprathreshold, and submaximal). Data are shown for the dorsal hippocampus (**A**–**C**) and the ventral hippocampus (**D**–**F**). Statistically significant differences are denoted by asterisks (independent *t*-test, ** *p* < 0.01).

Figure 6 and Figure 7 show the results of frequency stimulation on the steady state response, i.e., fEPSP 8–10. We detected significant differences related to sex, region, and stimulation intensity.

Specifically, performance to frequency stimulation is expressed with a greater frequency depression of steady state synaptic responses in females at relatively higher stimulation frequencies and increased facilitation of synaptic responses in males at intermediate stimulation frequencies. Significant sex-related differences were detected in the dorsal hippocampus at suprathreshold stimulation (UNIANOVA, *F*_(1, 263)_ = 5.9, *p =* 0.016), but not subthreshold (UNIANOVA, *F*_(1, 252)_ = 1.2, *p* = 0.275) or submaximal stimulation (UNIANOVA, *F*_(1, 238)_ = 0.51, *p* = 0.476). In the ventral hippocampus, we detected significant differences between females and males at subthreshold (UNIANOVA, *F*_(1, 263)_ = 21.76, *p* < 0.001) but not suprathreshold (UNIANOVA, *F*_(1, 275)_ = 0.04, *p* = 0.839) or submaximal stimulation intensities (UNIANOVA, *F*_(1, 251)_ = 2.04, *p* = 0.155).

### 2.3. Short-Term Neuronal Dynamics (STND) Differ Between Female and Male Hippocampus

**Figure 8:**
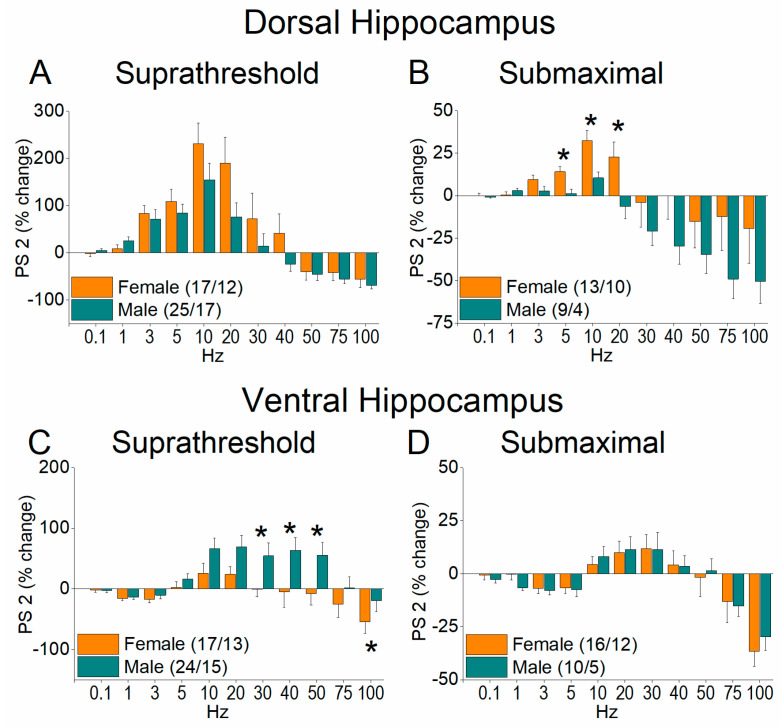
Frequency-dependent modulation of PS in the dorsal hippocampus (**A**,**B**) and ventral hippocampus (**C**,**D**) of female and male rats. Results for the first conditioned response (PS 2, onset response) are presented under two stimulation intensities: suprathreshold, and submaximal. Statistically significant differences are denoted by asterisks (independent *t*-test, * *p* < 0.05). Note the region-specific response to frequency stimulation in the onset response between the two sexes under suprathreshold stimulation: frequency stimulation induces greater facilitation in the dorsal hippocampus of females and the ventral hippocampus of males.

**Figure 9:**
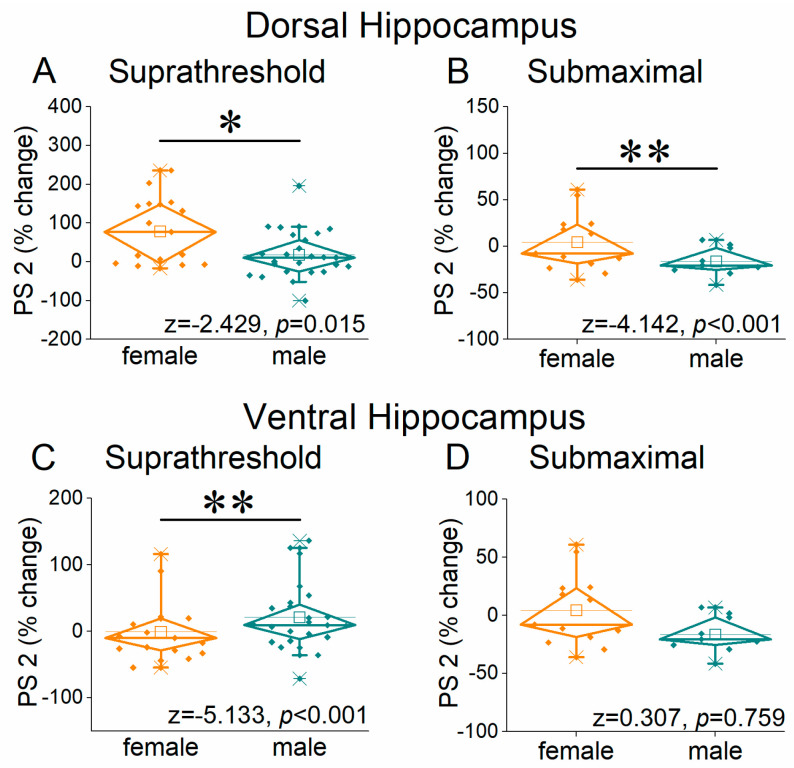
The average percent effects of frequency stimulation on PS 2 are shown for the female and male dorsal hippocampus (**A**,**B**) and ventral hippocampus (**C**,**D**). Statistically significant differences are denoted by asterisks (independent *t*-test, * *p* < 0.05, ** *p* < 0.01).

We next examined neuronal excitation by assessing population spikes (PS) recorded from the stratum pyramidale under suprathreshold conditions, producing a PS of 1.0–2.0 mV, and submaximal condition, producing a PS of 75% of its maximum amplitude. A unified picture emerges from these measurements that differ from that concerning synaptic transmission. Specifically, in contrast to the lower facilitation of fEPSP 2 seen in the female compared with the male hippocampus, both dorsal and ventral, the first conditioned response (onset response, PS 2) of the neuronal output increased in the dorsal female hippocampus, but in the ventral hippocampus, it increased in males (Figure 8 and Figure 9, and Supplementary Figures S3 and S4). In the dorsal hippocampus, we found higher facilitation of PS 2 in females than in males at both the suprathreshold stimulation intensity (UNI-ANOVA, *F*_(1, 320)_ = 8.98, *p* = 0.003) and submaximal stimulation intensity (UNIANOVA, *F*_(1, 211)_ = 14.48, *p* < 0.001) (Figure 8A,B and Figure 9A,B). In contrast, in the ventral hippocampus, we found increased facilitation of PS 2 in males than in females at the suprathreshold stimulation intensity (UNIANOVA, *F*_(1, 302)_ = 23.06, *p* < 0.001) but not at the submaximal stimulation intensity (UNIANOVA, *F*_(1, 261)_ = 0.003, *p* = 0.953) (Figure 8C,D and Figure 9C,D).

**Figure 10:**
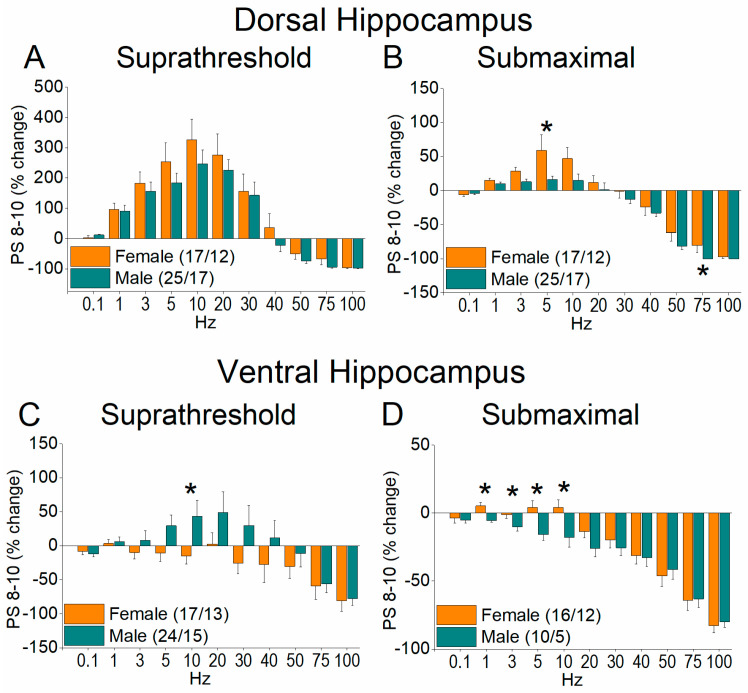
Frequency-dependent modulation of PS in the dorsal hippocampus (**A**,**B**) and ventral hippocampus (**C**,**D**) of female and male rats. Results for the steady state conditioned response (PS 8–10) are presented across two stimulation intensities: suprathreshold, and submaximal. Statistically significant differences are denoted by asterisks (independent *t*-test, * *p* < 0.05). Note that the ventral hippocampus of male rats exhibits greater frequency facilitation under suprathreshold stimulation and greater frequency depression under submaximal stimulation, suggesting a broader dynamic range of responses compared to the ventral hippocampus of female rats.

**Figure 11:**
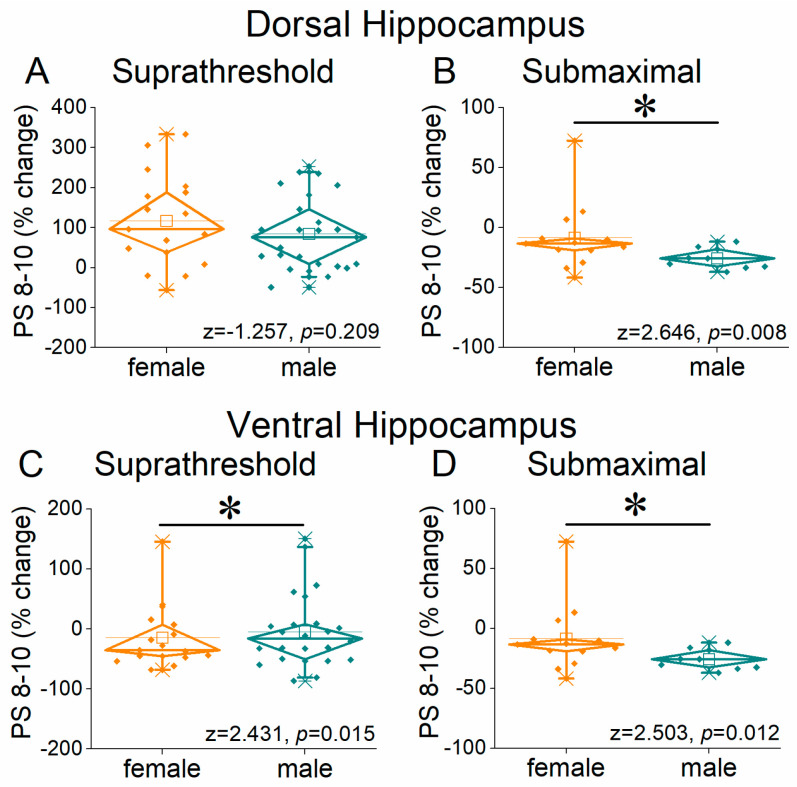
The average percent effects of frequency stimulation on fEPSP 8–10 are shown for both dorsal hippocampus (**A**,**B**) and ventral hippocampus (**C**,**D**) of females and males. Statistically significant differences are denoted by asterisks (independent *t*-test, * *p* < 0.05).

A similar boosting of the neuronal output was also seen for steady state responses (PS 8–10) in the female vs. male hippocampus (Figure 10 and Figure 11). More specifically, in the dorsal hippocampus, we found higher facilitation of PS 8–10 in females than males at both suprathreshold (UNI-ANOVA, *F*_(1, 323)_ = 4.38, *p* = 0.037) and submaximal stimulation intensities (UNIANOVA, *F*_(1, 211)_ = 11.82, *p* < 0.001) (Figure 10A,B and Figure 11A,B). As with PS 2, the changes in the steady state response were reversed in the ventral hippocampus, where we found greater facilitation of PS 8–10 in males than in females at the suprathreshold stimulation intensity (UNIANOVA, *F*_(1, 302)_ = 11.19, *p* < 0.001) and submaximal stimulation intensity (UNIANOVA, *F*_(1, 261)_ = 7.75, *p* = 0.006) (Figure 10C,D and Figure 11C,D).

## 3. Discussion

The main findings of the present study were: (1) females display lower frequency facilitation and increased frequency depression of synaptic transmission (fEPSP) in both segments of the hippocampus; (2) in contrast, a more complicated pattern of changes has been seen in STND, where increased frequency facilitation of neuronal excitation (PS) has been seen in the female dorsal hippocampus, and the male ventral hippocampus with suprathreshold but not submaximal stimulation.

### 3.1. Possible Interpretations

Specific properties of the female and male hippocampal circuit may offer explanations of these differences in short-term dynamics between the two biological sexes. For instance, a previous study using cell cultures has shown that the size of the recycling pool is larger and more frequently used in male than female synapses [56]. A slow and/or small recycling pool should be associated with insufficiently readily releasable pool, leading to a limited ability for short-term synaptic facilitation and increased tendency for short-term synaptic depression since the recovery from depression depends on replenishment kinetics, which are governed by the availability and mobilization of recycling vesicles [57,58,59]. Conversely, a relatively fast and/or large recycling pool can sustain high-frequency activity more effectively, such as in the present study, since synapses depend heavily on recycling pools to maintain neurotransmitter output [57,58,59]. Therefore, a less effective recycling pool in the female hippocampus could reliably explain the lower scores of frequency facilitation and greater frequency of the depression of synaptic responses observed in females compared to males in this study.

The present results are in apparent discrepancy with those of a recent study, which examined STSP in the male and female rat hippocampus in a model of Fragile X syndrome [60]. That study did not find significant differences in paired-pulse ratio between the male and female dorsal or ventral hippocampus. The observed discrepancies in STSP profiles may arise from several sources. Most notably, the current study did not control for the estrous cycle, which is known to significantly affect measures of synaptic transmission, neuronal excitability, and structural plasticity [17,19,61]. Estrogen fluctuations during the estrous cycle can markedly alter GABAergic inhibition and glutamatergic transmission [10,57,58], potentially masking or inflating subtle sex differences in STSP. Another explanation for this discrepancy may lie in strain-related differences, as the previous study used Long Evans, while Wistar rats were used in the present study. Differences in animal strain can influence plasticity-related properties, as strain-specific variations impact synaptic protein expression, dendritic architecture, and responses to hormonal status.

The present study is the first to systematically examine STND across both sexes and the dorsoventral axis of the hippocampus, revealing a novel, previously unappreciated functional asymmetry in how neuronal output is modulated during repetitive stimulation. The observed increased frequency in the facilitation of PS responses in the dorsal hippocampus of females and the ventral hippocampus of males suggests the existence of sex-related adaptations in network responsiveness, possibly associated with distinct cognitive and/or emotional processing properties in females and males. Mechanistically, these STND differences may arise from sex- and region-specific variations in synaptic inhibition and excitatory drive. It has been recently shown that inhibition plays a crucial role in shaping the properties of short-term changes in neuronal firing during high-frequency short bursts of activity [46]. Specifically, reduced inhibition leads to higher scores of frequency facilitation and lower frequency depression of PS [46].

Previous studies have documented sex-related differences in GABAergic inhibition with estrogens reducing GABAergic inhibition in the hippocampus of adult female rats, leading to increased excitability [57,58]. Furthermore, the expression of estrogens appears higher in the dorsal than ventral hippocampus, especially in GAD-positive interneurons in females [28], suggesting an estrogen-mediated disinhibition mechanism in females. This could explain the enhanced dorsal STND facilitation in females, and its ventral shift in males. Furthermore, substantial sex-related differences in dendritic structure, such as higher dendritic spine density and branching in the female hippocampus, especially during proestrus [32,36], and regional variance in dendritic complexity, could contribute to the observed regional differentiation in STND. These morphological features may shape the integration and propagation of repetitive synaptic inputs across the hippocampal circuit in a sex- and region-specific manner.

### 3.2. Implications

Short-term dynamics are critical for the encoding, amplification, gating, and filtering of incoming signals in a timescale of milliseconds to seconds [1,2,3,4,5,43,62]; neural processes linked to various brain functions, such as working memory, attentional gating, and emotional processing, are known to exhibit sex-specific profiles. Therefore, the observed sex- and region-dependent differences in STSP and STND may reflect distinct modes of hippocampal information processing and have important implications for such brain functions between females and males, possibly conferring distinct computational advantages or vulnerabilities relevant to each sex.

In addition, considering the functional segregation along the dorsoventral hippocampal axis, with the dorsal segment primarily engaged in cognitive tasks, such as spatial memory and attention, and the ventral segment more involved in affective processing and stress regulation [54,55,63], our findings suggest that sex differences in short-term dynamics may be specifically related to sex-specific cognitive and affective functions. For instance, enhanced short-term facilitation of neuronal output in the dorsal hippocampus of females may support sex-specific cognitive flexibility or working memory processing, e.g., optimizing short-term encoding in spatial memory tasks, while greater facilitation in the ventral hippocampus of males may alter emotional reactivity or stress responsiveness.

Furthermore, these physiological distinctions may underlie known sex biases in the prevalence and presentation of neuropsychiatric disorders. For instance, in autism spectrum disorder, which is typically more prevalent in males [64], altered short-term dynamics have been implicated in abnormal sensory filtering and social cognition [52,53,60], and males display higher susceptibility to alterations in synaptic plasticity than females [65]. Hence, sex-specific hippocampal dynamics could underlie differential vulnerability and phenotypic expression [11]. In contrast, depression, which is more prevalent in females and linked to dysfunction in the ventral hippocampus [66,67], may be exacerbated by reduced excitatory dynamics and enhanced synaptic and neuronal depression observed in female ventral CA1 circuits.

In line with our findings, sex-dependent adaptations of the glutamatergic system may provide an important mechanism for these differences in short-term dynamics. The glutamate system is fundamental for hippocampal neuroplasticity, and accumulating evidence indicates that it is differentially regulated in females and males. For instance, under physiological conditions, sex differences in glutamate release, receptor composition, and plasticity have been reported in cortical, limbic, and reward-related regions [12], suggesting that excitatory signaling is adapted in a sex-specific manner to support distinct modes of synaptic integration and behavior. In pathophysiological contexts, these differences may be related to different vulnerabilities. For instance, acute stress alters AMPA/NMDA receptor subunits and impairs recognition memory in a sex-dependent fashion [68]. Thus, sex-specific regulation of glutamatergic signaling may underlie, at least in part, the region- and sex-dependent differences in STSP and STND observed here, while also contributing to sex-biased risk for neuropsychiatric disorders. Future research should aim to identify the molecular and circuit-level mechanisms underlying these sex-related differences in short-term dynamics. In addition to glutamatergic transmission, including synaptic proteins and postsynaptic receptors, emphasis could also be placed on hormonal modulation, and neuronal subtype-specific contributions.

## 4. Materials and Methods

### 4.1. Animals and Hippocampal Slice Preparation

Transverse hippocampal slices (550 μm thick) were obtained from 3–4-month-old female and male Wistar rats housed in the Laboratory of Experimental Animals, Department of Medicine, University of Patras (license: EL-13-BIOexp-04). Animals were maintained at a stable temperature (20–22 °C) and under a 12:12 h light-dark cycle with ad libitum access to food and water. All procedures complied with EU Directive 2010/63/EU and were approved by institutional and regional authorities (reg. no. 187531/626, 26 June 2018). Following deep anesthesia with diethyl ether and decapitation, brains were quickly removed and placed in ice-cold oxygenated artificial cerebrospinal fluid (ACSF). Slices were prepared from the dorsal and ventral hippocampus (0.5–3.5 mm from each end) and transferred to an interface chamber perfused with ACSF, containing, in mM: 124 NaCl, 4 KCl, 2 CaCl_2_, 2 MgSO_4_, 26 NaHCO_3_, 1.25 NaH_2_PO_4_, and 10 glucose (Merck KGaA, Darmstadt, Germany), equilibrated with 95% O_2_ and 5% CO_2_ gas mixture (Chrysanthakopouloi A. & S. O.E., Patra, Greece) at pH = 7.4, at 30 ± 0.5 °C. Slices were continuously humidified with a mixed gas consisting of 95% O_2_ and 5% CO_2_. After at least 90 min of recovery, electrophysiological recordings were initiated.

### 4.2. Electrophysiology and Data Acquisition

The field potential was recorded in the middle CA1 region following stimulation of the Schaffer collaterals. A bipolar platinum/iridium electrode (25 μm wire diameter, 100 μm inter-wire spacing; WPI, Worcester, MA, USA) delivered constant current pulses (100 μs duration, 20–260 μA). Field excitatory postsynaptic potentials (fEPSPs) and population spikes (PS) were recorded in the stratum radiatum and stratum pyramidale, respectively, using 7 μm carbon fiber electrodes (Kation Scientific, Minneapolis, MN, USA) placed ~350 μm from the stimulation site. Recordings often captured both fEPSP and PS simultaneously. Signals were amplified ×500, filtered (0.5 Hz–2 kHz), digitized at 10 kHz, and stored for offline analysis (CED 1401-plus and Signal5.9 software, Cambridge Electronic Design, Milton, UK).

### 4.3. Stimulation Protocols and Quantification

Input–output curves were constructed to determine stimulation levels, and baseline pulses were applied every 30 s using current intensities that elicited near-threshold fEPSPs. Since stimulation intensities could vary between slices, the stimulation scale was expressed as a percentage of the maximum intensity used to construct the I–O curve in each slice. More specifically, the stimulation scale was divided into ten intervals after determining the intensities that elicited the minimal and maximal responses [69]. For short-term synaptic plasticity (STP) analysis, we employed a frequency stimulation protocol comprising ten-pulse trains delivered at frequencies ranging from 0.1 to 100 Hz. This pattern mimics naturally occurring spike trains in hippocampal pyramidal neurons [70]. Trains were applied in random order, with 2-min inter-train intervals to ensure synaptic recovery. To study activity-dependent changes, stimulation was applied at three defined intensities: subthreshold: eliciting ~0.5 mV/ms fEPSP without PS; suprathreshold: evoking a PS of 1.0–2.0 mV; submaximal: evoking a PS at ~75% of maximal amplitude [46,71]. STP of fEPSP and PS was quantified by measuring the percent change of each of the nine conditioned responses (2nd–10th pulses) relative to the first (conditioning response). Steady state responses were defined as the average of the 8th–10th pulses. The fEPSP slope was measured within 1 ms of the fiber volley onset, and PS amplitude was calculated as the vertical projection of the minimum peak relative to the line connecting the two flanking positive peaks.

### 4.4. Statistical Analysis

Comparisons between two groups were performed using the independent *t*-test. The assumption of equal variances and normal distribution of data was evaluated using Levene’s test and the Shapiro–Wilk test, respectively. The univariate full factorial (UNIANOVA) general linear model (GLM) with fixed-effect factors (i.e., regression analysis and analysis of variance for one dependent variable) was used to compare STSP and STND between the two sexes. Each hippocampal slice was considered an independent experimental unit. Data are expressed as the mean ± S.E.M. In column graphs, values are presented as the mean ± S.E.M., while box plots depict the median and interquartile range (25th to 75th percentiles; diamond-shaped box), mean (thick line), 5th and 95th percentiles (whiskers), and individual data points (superimposed). The number of slices and animals used (expressed as slices/animals) are reported throughout the text. Statistical analyses were performed based on the number of slices. All analyses were conducted using IBM SPSS Statistics version 27.

## Data Availability

All data associated with this study are available from the corresponding author upon reasonable request.

## Supplementary Materials

The supporting information can be downloaded at: https://www.mdpi.com/article/10.3390/ijms26178424/s1.

## Abbreviations

ACSF, artificial cerebrospinal fluid; fEPSP, field excitatory postsynaptic potential; PS, population spike, STSP, short-term synaptic plasticity; STND, short-term neuronal dynamics.
